# Nanomedicine and Its Role in Surgical Wound Infections: A Practical Approach

**DOI:** 10.3390/bioengineering12020137

**Published:** 2025-01-31

**Authors:** Malak Bentaleb, Mohammed Abdulrahman, Marcelo A. F. Ribeiro Jr

**Affiliations:** 1 College of Medicine and Health Sciences, Khalifa University, Abu Dhabi P.O. Box 127788, United Arab Emirates; 100062666@ku.ac.ae (M.B.); 100062598@ku.ac.ae (M.A.); 2 R Adams Cowley Shock Trauma Center, University of Maryland School of Medicine, Baltimore, MD 21201, USA

**Keywords:** nanoparticles, wound healing, wound infection, lipid nanoparticles, metal nanoparticles, polymer nanoparticles

## Abstract

Surgical wound infections are a major cause of postoperative complications, contributing to surgical morbidity and mortality. With the rise of antibiotic-resistant pathogens, it is crucial to develop new innovative wound materials to manage surgical wound infections using methods that facilitate drug delivery agents and rely on materials other than antimicrobials. Nanoparticles, in particular, have captured researchers’ interest in recent years due to their effectiveness in wound care. They can be classified into three main types: inorganic nanoparticles, lipid-based nanoparticles, and polymeric nanoparticles. Several studies have demonstrated the effectiveness of these new technologies in enhancing wound-healing times and reducing bacterial burden. However, further research is essential to thoroughly evaluate the safety and toxicity of these materials before they can be integrated into routine surgical practice.

## 1. Introduction

Surgical wound infections are some of the most reported forms of nosocomial infections with significant effects on morbidity and mortality [1]. Most recent data state that surgical wound infections account for over 2 million nosocomial infections in the United States and are associated with 11% of ICU deaths [1]. Surgical site infections are associated with increased hospital stays and a financial burden estimated at more than USD 20,000 per admission [2]. The primary sources of microbes causing surgical wound infections are the skin and adjacent tissue at the surgical site, as well as more internal structures associated with the procedure [3]. Some of the most commonly isolated microorganisms are *Staphylococcus aureus*, coagulase-negative *Staphylococci*, *Escherichia coli*, *Enterococcus faecalis*, and *Pseudomonas aeruginosa* [4]. In the presence of rising antimicrobial resistance rates across the world [5], there is a serious need to develop novel ways to tackle this ongoing global health crisis. Nanotechnology has limitless capacities to advance and aid a plethora of different medical applications, including, but not limited to, cancer, tissue repair, imaging techniques, gene therapy, and more [6,7,8]. The US Food and Drug Administration (FDA) and World Health Organization (WHO) have already approved several nanodrugs and nanoparticle polymers that may potentially be used as antimicrobials [9,10].

Nanotechnology refers to the manipulation and use of matter on the nanoscale, typically ranging from 1–100 nm. Nanoparticles exhibit unique properties due to the scale at which they operate, such as biological mobility and chemical reactivity, making them different even from that of their bulky counterparts [11,12]. One of the main properties that allows for nanoparticles’ unique chemical behaviors is their large surface area [13]. At this scale, these nanoparticles obey the laws of quantum mechanics as opposed to the traditional laws of physics and chemistry that govern larger-scale systems [14]. In essence, this combination of properties allows for nanoparticles to be manipulated and used in unique ways that take advantage of their size and surface area to enhance a variety of applications in medicine.

Within the context of infection, metal-based nanoparticles hold great promise in the treatment of bacterial infections due to their antibacterial activity [15]. A few of the most crucial factors for the efficacy of metal-based nanoparticles are their high surface area-to-volume ratio, size, and bioavailability [16,17]. Nanoparticles are smaller than bacterial cells and can therefore diffuse into the cell and exhibit their antibacterial properties via a variety of mechanisms, including membrane potential disruption and generation of reactive oxygen species [18,19]. These basic principles lay the foundation for how nanoparticles are used in the context of surgical wound infections. The main objective of this review is to provide clinicians and surgeons a better understanding of the real-world application and use of nanoparticles in the treatment of surgical wound infections. Therefore, particular interest will be given to the practical use of nanomedicine as an adjunct or replacement to typical antimicrobials, in both future and current surgical practice. Scrutiny of nanomedicine’s practicality, drug delivery mechanisms, success rates, future implications, cost, complications, indications, and contraindications is crucial for the viable adoption of nanomedicine into routine clinical practice.

## 2. Role of Nanotechnology in Surgical Wound Infection

Surgical wound infections are a major cause of postoperative complications, contributing to surgical morbidity and mortality. Recent data shows that surgical wound infection represents a significant number of hospital infections [1]. The prevalence of resistant nosocomial microorganisms, including methicillin-resistant S. aureus (MRSA) and extended-spectrum beta-lactamase bacteria, has been increasing recently [1]. The rise of antibiotic-resistant pathogens has become an emerging medical problem [20]. Therefore, it is crucial to develop new innovative wound materials to manage surgical wound infections using methods that facilitate drug delivery agents and rely on materials other than antimicrobials.

Nanotechnology has emerged as an effective approach to enhance wound healing. Nanoparticles, in particular, have captured researchers’ interest in recent years due to their effectiveness in wound care. They can be classified into three main types: inorganic nanoparticles, lipid-based nanoparticles, and polymeric nanoparticles [21]. We will explore the use of each one of these new technologies in wound healing and antimicrobial activity. Table 1 represents the mechanisms, advantages, and disadvantages of the different types of nanoparticles discussed within this paper.

## 3. Inorganic Nanoparticles

### 3.1. Metal Based Inorganic Nanoparticles

Several studies have investigated the efficacy of nanoparticles with regards to antimicrobial properties and infection, both in vivo and in vitro. Nanoparticle-based drugs use a variety of compositions, including metal, lipid, carbon, and polymeric-based nanoparticles [22]. Silver (Ag) and gold (Au)-based metal nanoparticles are some of the most commonly investigated and used [22].

Inorganic nanoparticles derived from metals, including silver, copper, zinc, gold, and others, are being extensively investigated due to their antimicrobial properties that are different from antibiotics [23]. Their antibacterial mechanism of action is still not yet fully understood, but some of the mechanisms described in the literature include the release of ions that can bind to thiol DNA bases and inhibit DNA replication [24], the inhibition of essential enzymes such as DNA polymerase [25] and beta-galactosidase [26], the disruption of the cellular membrane by causing photocatalytic reactions and increasing the membrane permeability [27,28,29], and the reduction of biofilm formation [23,30,31].

Metal nanoparticles have been shown to have synergetic effects when used with antibiotics and other biomaterials by increasing the delivery and effectiveness of the drug complex as well as reducing side effects [20,23]. They also allow for a slow, gradual, and sustained release of the antibiotic [32]. Metal nanoparticle drug complexes have various formulations and can be delivered through systemic, oral, or transdermal routes [23]. Many studies have investigated metal and polymeric nanoparticles and their use in potential wound dressings against different bacterial strains [33,34,35]. Metal nanoparticles can also potentially be used in the coating of medical implant materials to decrease post-surgical implantation infection risk [36,37]. More research is still needed to fully understand the efficacy of these promising systems.

Silver nanoparticles are the most commonly used and were among the first to be introduced clinically [38]. This is due to their strong antibacterial capabilities and their ability to address antibiotic resistance, which has been attributed to the reaction of silver ions with thiol groups of important enzymes [39]. Additionally, silver is known to have low toxicity to human cells [40].

A recent randomized clinical trial compared the use of kadermin, a silver nanoparticle-based cream, against mupirocin and showed significant differences in wound healing and bacteria clearance of culture-positive infected wounds [41]. Another randomized comparative trial used acticoat, a silver nanocrystalline wound dressing, for use in burn wounds, and the results showed significantly faster healing times and higher bacterial clearance when compared to the control [42]. In vitro biofilm production by *Pseudomonas aeruginosa* and *Staphylococcus epidermidis* was able to be significantly inhibited when treated with silver-based nanoparticles [30]. In another randomized control study comparing the efficacy of silver-based nanoparticle gel against traditional wound dressing in the management of nonischemic diabetic foot ulcer, results showed significantly faster wound-healing rates for the silver nanoparticle group [43].

While these findings are promising, notable differences in the employed methodologies and outcome measures indicate the need for large-scale standardized trials before integrating metal-based nanoparticles in clinical practice. For example, some studies focus on specific wound types such as ulcers and burns, while other studies report more generalized wound types, which makes direct comparison of the findings difficult. Additionally, the nanoparticle formulations and application techniques differ between studies, with some using topical formations and others wound dressings, which may influence the reported outcomes. Studies documenting outcomes for each wound context and nanoparticle application technique are needed to make direct comparisons.

Among metal nanoparticles, gold nanoparticles are also favored ones because of their biocompatibility as well as their low toxicity [44]. Gold nanoparticles have effective antibacterial properties through various mechanisms involving blocking DNA transcription and ATP synthesis, as well as increasing free radical activity [23,45]. Studies have shown that chitosan–gold nanoparticles significantly increased wound hemostasis and repair [46]. Hence, gold nanoparticles also present an encouraging approach in managing surgical wound infections.

While metal nanoparticles offer a new potential way of managing site infections, it is important to consider their potential risks. Due to their very small size, metal nanoparticles can readily penetrate cell membranes and build up in organelles, causing detrimental toxicity, genotoxicity, neurotoxicity, immunotoxicity, and carcinogenicity through various signaling cascades [47,48]. The safety of these nanoparticles still needs further research before they can be used in clinical practice.

Despite these promising results, there is still a lack of general consensus for when nanoparticle-based solutions should be implemented clinically. Additionally, many of the current clinical trials have been tested in small, specific populations, which limits the studies’ reproducibility in the real world. Nevertheless, the current documented efficacy of nanotechnology-based medicine in the real world is present and ever growing, with more and more interest being paid to the field of nanomedicine. With more rigorous studies validating their clinical efficacy, metal-based nanoparticle wound dressings could potentially replace traditional wound dressings, and topical creams and gels containing metal-based nanoparticles may be introduced as potential primary or adjunct treatments in post-surgical care.

### 3.2. Non-Metal Inorganic Nanoparticles

Non-metal inorganic nanoparticles that have been investigated in wound healing include carbon nanotubes and bioactive glass nanoparticles [49,50].

There are two types of carbon nanotubes: single-walled and multi-walled carbon nanotubes [51]. Several studies have shown the capacity of carbon nanoparticles in improving wound healing. Their mechanism of action is determined by their physical, chemical, and size properties [52,53]. While many studies have investigated the antibacterial and anti-fungal effects of carbon nanotubes, the exact mechanisms for their antimicrobial properties are still not well understood [52]. This may be attributed to the unavailability of standardized tests to evaluate antimicrobial properties [52]. They can inhibit bacterial proliferation by hindering the energy metabolism and disrupting the respiratory chain, which induces bacterial cell death [49]. Carbon nanotubes can generate reactive oxygen species through chemical interactions with the surface of microbes [53]. Additionally, some carbon nanomaterials can isolate cells from their environment, resulting in their possible death as well [53].

A study designed a non-antibiotic adhesive nanocomposite made of N-carboxyethyl chitosan (CEC) and benzaldehyde-terminated Pluronic F127/carbon nanotubes (PF127/CNT) [54]. They showed remarkable treatment results in full-thickness infected wounds in vivo with enhanced wound closure and angiogenesis [54]. A recent study investigated the effects of single-wall and multi-wall carbon nanotubes complexed with chitosan on full-thickness wound healing in a mouse model [55]. They both significantly improved tissue epithelization; however, increased fibrosis and inflammation were noted, especially in the multi-walled carbon nanotubes [55]. Therefore, further investigation is needed to explore the possible side effects of these technologies in tissue scarring and repair.

Both studies investigated the effects of carbon nanotube-based hydrogels on wound healing in full-thickness-induced wounds using mouse models. Both studies have similar methodologies and report positive outcomes, making carbon nanotube-based hydrogels potential candidates for wound healing in the future. If future clinical trials confirm their efficacy and validity, these hydrogels could be used to engineer new adhesives that can potentially serve as novel adhesives or adjuncts to the surgical adhesives used currently in practice. However, more research is needed to investigate the effect of these carbon nanotube-based hydrogels on bacterial clearance and potential tissue changes, such as fibrosis and tissue changes, to ensure their safety and efficacy.

Bioactive glass nanoparticles also present a promising way of managing surgical wounds. They improve wound healing by releasing ions that advance angiogenesis, M1-to-M2 macrophage switching, and antibacterial action [50]. Some studies showed that some bioactive glass compounds, such as S53P4, can suppress the growth of various bacteria, including MRSA, by creating holes in the cell membrane and changing its conformation [56]. A study comparing the antimicrobial activity of different bioactive glass compounds revealed that particles with smaller sizes exhibited a stronger antimicrobial activity [56]. In a recent study, bioactive glass nanoparticles functionalized with polydopamine were tested for infected wounds and skin tumor therapy [57]. It demonstrated exceptional wound healing and antibacterial effectiveness, including against the multidrug-resistant bacteria [57].

Several other studies explored the therapeutic effects of different bioactive glass-based biomaterials. However, comparing different materials is difficult due to the differences of models and the lack of standardized outcome measurements [50]. It becomes difficult to make direct comparisons without consistent criteria for assessing wound healing and bacterial clearance across different materials. Hence, further research is needed to identify the ideal model properties for clinical use.

## 4. Lipid Nanoparticles

Lipid nanoparticles are targeted drug delivery systems made of a biodegradable and biocompatible lipid matrix encased in a surfactant film [58]. They have the ability to contain various hydrophobic and hydrophilic agents, such as growth factors and gene therapy, and they allow for controlled, site-specific release of the therapeutic agent in damaged tissues with poor permeability [58,59]. Due to the fatty nature of solid lipid nanoparticles, they can allow for a slow release of the loaded antimicrobial, resulting in increased bacterial inhibitory effects and enhanced wound healing [60].

Lipid nanoparticles are perhaps most famously known for their use in the COVID-19 vaccines, for which they were crucial in the effective delivery of the mRNA vaccine content [61]. Unlike metal nanoparticles, which are used for their natural antimicrobial properties [62], lipid nanoparticles are mostly investigated for their effectiveness in drug delivery [63]. Drug delivery advancements are crucial in the control of microbes, as they can potentially be used to overcome mechanisms of antimicrobial resistance such as biofilm production [64].

There are different types of lipid nanoparticles, including liposomes, solid lipid nanoparticles, nanostructured lipid carriers, and more [58]. Lipid-based nanoparticles have been approved for use in various fields, including cancer therapy, infectious diseases, and anesthesia [65,66]. In the context of surgical wound infections, they hold significant potential. A recent study explored the efficacy of lipid–polymer hybrid nanoparticles combined with fusidic acid in combating resistant bacterial infections in burn wounds [67]. Murine burn wound models dispensed with MRSA were used to assess the potential of the lipid–polymer-based nano-engineered system in reducing the infection in vivo [67]. The results showed a significant decrease in MRSA bacterial burden as well as an enhanced wound size reduction in the treated group [67]. Hence, lipid-based nanoparticles offer an encouraging option in managing antimicrobial-resistant bacterial infections. Zhou et al. evaluated the in vivo antibacterial effect of ferric-loaded lipid nanoparticles on S. aureus-infected wound models [68]. The wounds treated with the ferric-loaded lipid nanoparticles showed remarkably reduced skin infections and enhanced wound healing [68]. Another study investigated the use of a solid lipid nanoparticle with the antibacterial lacticin 3147 hydrogel as a topical treatment for *S. aureus* wound infections [69]. Compared to free lacticin, the use of the solid lipid nanoparticle gel showed activity for a longer period of time (11 days vs. 9 days) [69]. While lipid nanoparticles investigated in these studies promote wound healing and bacterial eradication, it is important to note that they have been used as carriers for different materials. In one study, lipid nanoparticles served as a carrier platform for an antibiotic (fusidic acid) [67], while in another, they were used to deliver iron [68], and yet in another, they were used to deliver a bacteriocin [69]. This difference is important to highlight as the therapeutic effects observed in each study may be influenced not only by the lipid nanoparticles themselves but also by the specific substances they are transporting. Lipid-based nanotechnology holds significant potential for synergetic action with other antimicrobial agents in combating wound infections. Additionally, they may offer the possibility of reduced drug administration and treatment costs [58]. In the future of post-surgical care, topical creams and gels containing antimicrobial-loaded lipid nanoparticles may be introduced as primary or adjunct therapies.

While lipid nanoparticles have been used to great success in other medical applications (e.g., vaccine creation), they have yet to be fully utilized in the effort against antimicrobial resistance. More research needs to be done to establish support for eventual clinical trials to demonstrate the potential that lipid nanoparticles have in delivering antimicrobials and resolving antibiotic resistance infections.

## 5. Polymer Nanoparticles

Polymeric nanoparticles are also amongst the commonly studied nanoparticles. Polymeric nanoparticles function as robust drug carriers that can shield the drug from the environment and enhance their bioavailability [70]. Polymeric nanoparticles may also regulate the pharmacokinetics of many active substances [71]. They may be invaluable in the fight against surgical wound infections, as polymeric nanoparticles possess the capacity to increase drug bioavailability and enhance drug delivery at specific sites of action [72]. Polymeric materials have been shown to have potent antimicrobial properties [73], notably, polyhexamethylene biguanide (PHMB), which has been used in several settings, including wound dressing [74]. Polymeric nanoparticles are the most common type of nanoparticle within the FDA-approved nanoparticle drugs [75]. Many studies have demonstrated the efficacy of polymeric nanoparticles in combatting wound infections in vitro and in vivo. Hasan et al. created a clindamycin-loaded polymeric nanoparticle and tested its efficacy against MRSA wound infections [76]. The treated group showed a decrease in bacterial burden as well as accelerated wound healing [76]. These reported results show the potential of polymeric nanoparticles as another avenue to combat bacterial infection, creating new opportunities to combat antibiotic resistance.

Currently in the market, due to their biocompatibility and their accelerated wound healing, many polymeric materials such as chitosan are used and branded as wound dressings in the form of sponges, sprays, gauze, bandages, and patches [77]. One example of marketed polymer nanostructured materials is FibDex^®^, which is a nanofibrillar cellulose wound dressing that is effective in managing wounds [78]. Another example is Epiprotect^®^, which is a dressing made of biosynthetic cellulose in a nanostructure comparable to collagen, and it has been shown to be effective in treating wound burns in a pilot study for a pediatric population [79].

Additionally, many studies use polymer nanomaterials with metals and other conjugated nanomaterials for synergistic antimicrobial effects [80]. For example, an antimicrobial chitosan–hyaluronic acid and nano silver composite sponge was investigated as a potential future management of infected diabetic foot ulcers with drug-resistant bacteria [81]. The sponge demonstrated significant antibacterial activity against major wound bacteria, including MRSA [81].

Polymer nanoparticles are good candidates for applications in surgical wound healing; however, several challenges need to be addressed before being able to introduce them into clinical practice. These challenges include ensuring safety, enhancing the stability of polymeric nanoparticles, and creating reliable large-scale manufacturing techniques [20].

## 6. Limitations

There exist obstacles that need further scrutiny for nanoparticles to become more common in surgical practice. Namely, unintended immunological reactions place a key limit on the use of nanoparticles [82]. Nanoparticles may interact with the immune system and cause such effects as inadvertently activating the complement system and direct interaction with toll-like receptors, which may potentially contribute to immunotoxicity [82].

Additionally, it has been shown that certain nanoparticles possess significant cytotoxic effects despite their therapeutic properties. The cytotoxicity of different nanoparticles has been documented in several different environments which may limit their adoption in the clinical practice [83,84].

One study revealed that zinc oxide nanoparticles induced neurotoxicity and increased levels of inflammatory cytokines in the brain and the serum of treated mice as well as hippocampal pathological changes [85]. The same study revealed that the older the mouse, the more serious was the systemic inflammation [85]. Metal nanoparticles can also damage genetic material by entering the nucleus and engaging with DNA material [20].

Non-metal nanoparticle toxicity also poses a serious problem. Studies have shown that carbon nanotubes can have toxic effects on many organ systems as well as DNA [86]. Studies have shown that carbon nanotubes possess the capacity to cause DNA and chromosomal damage [87]. Other studies show that carbon nanosystems may enhance cytokine production and alter the immune system responses [88], as well as possibly increasing fibrosis [55]. Some studies demonstrate that certain carbon nanotube toxicities are dose dependent, with larger concentrations causing more severe side effects [86]. This highlights the need for careful study designs ensuring safe doses and concentrations before implementing these technologies into clinical use.

Liposomes can also have toxic side effects. Through interactions with biological components such as serum proteins, cationic liposomes can cause the release of loaded agents leading to systemic toxicity; these liposomes may also cause membrane poration and lysis [89]. Conjugating cationic liposomes with a targeting moiety can help mitigate certain risks such as liver damage caused by topical solutions [89]. Certain polymers may be used as surface protection to help reduce the toxic side effects of other nanoparticles [89].

## Figures and Tables

**Table 1:** Summary of Nanoparticle use in Surgical Wound Infection.

Nanoparticles in Surgical Wound Infection					
Nanoparticle Type	Metal Based	Carbon Based	Bioactive glass Based	Lipid Based	Polymer Based
Mechanism	- Inhibition of bacterial enzymes - Alteration of cell membrane	- Interruption of the respiratory chain - Impairing energy metabolism	- Improving wound healing via macrophage switching and advancing angiogenesis - Inhibiting bacterial growth through cell membrane disruption	- Enhanced delivery of therapeutic substances to site of action	- Regulation of pharmacokinetics - Improve bioavailability and drug delivery
Advantages	- Excellent antimicrobial properties - Improved wound healing - Established Randomized Control Trials conveying clinical effectiveness	- Improved wound closure - Improved angiogenesis	- Effective wound healing - Antimicrobial activity against drug resistant strains	- Ability to transport a variety of biological compounds - Synergistic potential with multiple antimicrobials - Successful application in other fields	- Effective carriers of drugs - Biocompatibility - Decrease in bacterial burden with resistant strains.
Disadvantages	- Potential immunotoxicity and genotoxicity	- Reported increase in fibrosis and inflammation - Lack of standardized studies	- Lack of standardized measures to assess outcomes of different bioactive glass materials	- Lack of standardized studies showing lipid based nanoparticles in clinical use in surgical wound infections.	- Lack of large scale manufacturing for polymeric nanoparticles

## Data Availability

The original contributions presented in this study are included in the article. Further inquiries can be directed to the corresponding author(s).

## References

[1] Zabaglo M., Leslie S.W., Sharman T. (2024). Postoperative Wound Infections. StatPearls.

[2] Zimlichman E., Henderson D., Tamir O., Franz C., Song P., Yamin C.K., Keohane C., Denham C.R., Bates D.W. (2013). Health Care–Associated Infections: A Meta-Analysis of Costs and Financial Impact on the US Health Care System. JAMA Intern. Med..

[3] Young P.Y., Khadaroo R.G. (2014). Surgical site infections. Surg. Clin. North Am..

[4] Sievert D.M., Ricks P., Edwards J.R., Schneider A., Patel J., Srinivasan A., Kallen A., Limbago B., Fridkin S., National Healthcare Safety Network T. (2013). Antimicrobial-Resistant Pathogens Associated with Healthcare-Associated Infections: Summary of Data Reported to the National Healthcare Safety Network at the Centers for Disease Control and Prevention, 2009–2010. Infect. Control Hosp. Epidemiol..

[5] Salam A., Al-Amin Y., Salam M.T., Pawar J.S., Akhter N., Rabaan A.A., Alqumber M.A.A. (2023). Antimicrobial Resistance: A Growing Serious Threat for Global Public Health. Healthcare.

[6] Contera S., Bernardino de la Serna J., Tetley T.D. (2020). Biotechnology, nanotechnology and medicine. Emerg. Top. Life Sci..

[7] Saini R., Saini S., Sharma S. (2010). Nanotechnology: The future medicine. J. Cutan. Aesthetic Surg..

[8] Malik S., Muhammad K., Waheed Y. (2023). Emerging Applications of Nanotechnology in Healthcare and Medicine. Molecules.

[9] Singh L., Kruger H.G., Maguire G.E., Govender T., Parboosing R. (2017). The role of nanotechnology in the treatment of viral infections. Ther. Adv. Infect. Dis..

[10] Makabenta J.M.V., Nabawy A., Li C.-H., Schmidt-Malan S., Patel R., Rotello V.M. (2021). Nanomaterial-based therapeutics for antibiotic-resistant bacterial infections. Nat. Rev. Microbiol..

[11] Murthy S.K. (2007). Nanoparticles in modern medicine: State of the art and future challenges. Int. J. Nanomed..

[12] Medina C., Santos-Martinez M.J., Radomski A., I Corrigan O., Radomski M.W. (2007). Nanoparticles: Pharmacological and toxicological significance. Br. J. Pharmacol..

[13] Joseph T.M., Mahapatra D.K., Esmaeili A., Piszczyk Ł., Hasanin M.S., Kattali M., Haponiuk J., Thomas S. (2023). Nanoparticles: Taking a Unique Position in Medicine. Nanomaterials.

[14] Sim S., Wong N.K. (2021). Nanotechnology and its use in imaging and drug delivery (Review). Biomed. Rep..

[15] Pang Q., Jiang Z., Wu K., Hou R., Zhu Y. (2023). Nanomaterials-Based Wound Dressing for Advanced Management of Infected Wound. Antibiotics.

[16] Nqoro X., Taziwa R. (2024). Polymer-Based Functional Materials Loaded with Metal-Based Nanoparticles as Potential Scaffolds for the Management of Infected Wounds. Pharmaceutics.

[17] Pan S., Goudoulas T.B., Jeevanandam J., Tan K.X., Chowdhury S., Danquah M.K. (2021). Therapeutic Applications of Metal and Metal-Oxide Nanoparticles: Dermato-Cosmetic Perspectives. Front. Bioeng. Biotechnol..

[18] Yudaev P., Mezhuev Y., Chistyakov E. (2022). Nanoparticle-Containing Wound Dressing: Antimicrobial and Healing Effects. Gels.

[19] Wang L., Hu C., Shao L. (2017). The antimicrobial activity of nanoparticles: Present situation and prospects for the future. Int. J. Nanomed..

[20] Sangnim T., Puri V., Dheer D., Venkatesh D.N., Huanbutta K., Sharma A. (2024). Nanomaterials in the Wound Healing Process: New Insights and Advancements. Pharmaceutics.

[21] Gowda B., Mohanto S., Singh A., Bhunia A., Abdelgawad M., Ghosh S., Ansari M., Pramanik S. (2023). Nanoparticle-based therapeutic approaches for wound healing: A review of the state-of-the-art. Mater. Today Chem..

[22] Huang Y., Guo X., Wu Y., Chen X., Feng L., Xie N., Shen G. (2024). Nanotechnology’s frontier in combatting infectious and inflammatory diseases: Prevention and treatment. Signal Transduct. Target. Ther..

[23] Altun E., Aydogdu M.O., Chung E., Ren G., Homer-Vanniasinkam S., Edirisinghe M. (2021). Metal-based nanoparticles for combating antibiotic resistance. Appl. Phys. Rev..

[24] Li R., Chen J., Cesario T.C., Wang X., Yuan J.S., Rentzepis P.M. (2016). Synergistic reaction of silver nitrate, silver nanoparticles, and methylene blue against bacteria. Proc. Natl. Acad. Sci. USA.

[25] Khan S.T., Malik A., Wahab R., Abd-Elkader O.H., Ahamed M., Ahmad J., Musarrat J., Siddiqui M.A., Al-Khedhairy A.A. (2017). Synthesis and characterization of some abundant nanoparticles, their antimicrobial and enzyme inhibition activity. Acta Microbiol. Et Immunol. Hung..

[26] Cha S.-H., Hong J., McGuffie M., Yeom B., VanEpps J.S., Kotov N.A. (2015). Shape-Dependent Biomimetic Inhibition of Enzyme by Nanoparticles and Their Antibacterial Activity. ACS Nano.

[27] Singh R., Cheng S., Singh S. (2020). Oxidative stress-mediated genotoxic effect of zinc oxide nanoparticles on Deinococcus radiodurans. 3 Biotech.

[28] Seong M., Lee D.G. (2017). Silver Nanoparticles Against Salmonella enterica Serotype Typhimurium: Role of Inner Membrane Dysfunction. Curr. Microbiol..

[29] Kiwi J., Nadtochenko V. (2005). Evidence for the Mechanism of Photocatalytic Degradation of the Bacterial Wall Membrane at the TiO_2_ Interface by ATR-FTIR and Laser Kinetic Spectroscopy. Langmuir.

[30] Kalishwaralal K., BarathManiKanth S., Pandian S.R.K., Deepak V., Gurunathan S. (2010). Silver nanoparticles impede the biofilm formation by Pseudomonas aeruginosa and Staphylococcus epidermidis. Colloids Surf. B Biointerfaces.

[31] Benoit D.S.W., Sims K.R., Fraser D. (2019). Nanoparticles for Oral Biofilm Treatments. ACS Nano.

[32] Assadi Z., Emtiazi G., Zarrabi A. (2018). Hyperbranched polyglycerol coated on copper oxide nanoparticles as a novel core-shell nano-carrier hydrophilic drug delivery model. J. Mol. Liq..

[33] Jatoi A.W. (2020). Polyurethane nanofibers incorporated with ZnAg composite nanoparticles for antibacterial wound dressing applications. Compos. Commun..

[34] Ashfaq M., Verma N., Khan S. (2016). Copper/zinc bimetal nanoparticles-dispersed carbon nanofibers: A novel potential antibiotic material. Mater. Sci. Eng. C.

[35] Altun E., Aydogdu M.O., Crabbe-Mann M., Ahmed J., Brako F., Karademir B., Aksu B., Sennaroglu M., Eroglu M.S., Ren G. (2019). Co-Culture of Keratinocyte-*Staphylococcus aureus* on Cu-Ag-Zn/CuO and Cu-Ag-W Nanoparticle Loaded Bacterial Cellulose:PMMA Bandages. Macromol. Mater. Eng..

[36] Rezk A.I., Sasikala A.R.K., Nejad A.G., Mousa H.M., Oh Y.M., Park C.H., Kim C.S. (2019). Strategic design of a Mussel-inspired in situ reduced Ag/Au-Nanoparticle Coated Magnesium Alloy for enhanced viability, antibacterial property and decelerated corrosion rates for degradable implant Applications. Sci. Rep..

[37] Mokabber T., Cao H.T., Norouzi N., van Rijn P., Pei Y.T. (2020). Antimicrobial Electrodeposited Silver-Containing Calcium Phosphate Coatings. ACS Appl. Mater. Interfaces.

[38] Zheng Q., Chen C., Liu Y., Gao J., Li L., Yin C., Yuan X. (2024). Metal Nanoparticles: Advanced and Promising Technology in Diabetic Wound Therapy. Int. J. Nanomed..

[39] Larrañaga-Altuna M., Zabala A., Llavori I., Pearce O., Nguyen D.T., Caro J., Mescheder H., Endrino J.L., Goel G., Ayre W.N. (2021). Bactericidal surfaces: An emerging 21st-century ultra-precision manufacturing and materials puzzle. Appl. Phys. Rev..

[40] Rai M., Yadav A., Gade A. (2009). Silver nanoparticles as a new generation of antimicrobials. Biotechnol. Adv..

[41] Pathi B.K., Mishra S., Moharana N., Kanungo A., Mishra A., Sahu S., Dash R.K., Dubey R., Das M.K. (2024). Effect of Topical Silver Nanoparticle Formulation on Wound Bacteria Clearance and Healing in Patients With Infected Wounds Compared to Standard Topical Antibiotic Application: A Randomized Open-Label Parallel Clinical Trial. Cureus.

[42] Huang Y., Li X., Liao Z., Zhang G., Liu Q., Tang J., Peng Y., Liu X., Luo Q. (2007). A randomized comparative trial between Acticoat and SD-Ag in the treatment of residual burn wounds, including safety analysis. Burns.

[43] Essa M.S., Ahmad K.S., Zayed M.E., Ibrahim S.G. (2023). Comparative Study Between Silver Nanoparticles Dressing (SilvrSTAT Gel) and Conventional Dressing in Diabetic Foot Ulcer Healing: A Prospective Randomized Study. Int. J. Low. Extrem. Wounds.

[44] Bansal S.A., Kumar V., Karimi J., Singh A.P., Kumar S. (2020). Role of gold nanoparticles in advanced biomedical applications. Nanoscale Adv..

[45] Rajendran N.K., Kumar S.S.D., Houreld N.N., Abrahamse H. (2018). A review on nanoparticle based treatment for wound healing. J. Drug Deliv. Sci. Technol..

[46] Hsu S.-H., Chang Y.-B., Tsai C.-L., Fu K.-Y., Wang S.-H., Tseng H.-J. (2011). Characterization and biocompatibility of chitosan nanocomposites. Colloids Surf. B Biointerfaces.

[47] Xiong P., Huang X., Ye N., Lu Q., Zhang G., Peng S., Wang H., Liu Y. (2022). Cytotoxicity of Metal-Based Nanoparticles: From Mechanisms and Methods of Evaluation to Pathological Manifestations. Adv. Sci..

[48] Zhang N., Xiong G., Liu Z. (2022). Toxicity of metal-based nanoparticles: Challenges in the nano era. Front. Bioeng. Biotechnol..

[49] Patil T.V., Patel D.K., Dutta S.D., Ganguly K., Randhawa A., Lim K.-T. (2021). Carbon Nanotubes-Based Hydrogels for Bacterial Eradiation and Wound-Healing Applications. Appl. Sci..

[50] Guerrero M.V., Aendekerk F.M.A., de Boer C., Geurts J., Lucchesi J., Arts J.J.C. (2024). Bioactive-Glass-Based Materials with Possible Application in Diabetic Wound Healing: A Systematic Review. Int. J. Mol. Sci..

[51] Baughman R.H., Zakhidov A.A., de Heer W.A. (2002). Carbon Nanotubes—The Route Toward Applications. Science.

[52] Moskvitina E., Kuznetsov V., Moseenkov S., Serkova A., Zavorin A. (2023). Antibacterial Effect of Carbon Nanomaterials: Nanotubes, Carbon Nanofibers, Nanodiamonds, and Onion-like Carbon. Materials.

[53] Al-Jumaili A., Alancherry S., Bazaka K., Jacob M.V. (2017). Review on the Antimicrobial Properties of Carbon Nanostructures. Materials.

[54] He J., Shi M., Liang Y., Guo B. (2020). Conductive adhesive self-healing nanocomposite hydrogel wound dressing for photothermal therapy of infected full-thickness skin wounds. Chem. Eng. J..

[55] Kittana N., Assali M., Abu-Rass H., Lutz S., Hindawi R., Ghannam L., Zakarneh M., Mosa A. (2018). Enhancement of wound healing by single-wall/multi-wall carbon nanotubes complexed with chitosan. Int. J. Nanomed..

[56] Zhou P., Garcia B.L., Kotsakis G.A. (2022). Comparison of antibacterial and antibiofilm activity of bioactive glass compounds S53P4 and 45S5. BMC Microbiol..

[57] Zhou L., Xi Y., Xue Y., Wang M., Liu Y., Guo Y., Lei B. (2019). Injectable Self-Healing Antibacterial Bioactive Polypeptide-Based Hybrid Nanosystems for Efficiently Treating Multidrug Resistant Infection, Skin-Tumor Therapy, and Enhancing Wound Healing. Adv. Funct. Mater..

[58] Motsoene F., Abrahamse H., Kumar S.S.D. (2023). Multifunctional lipid-based nanoparticles for wound healing and antibacterial applications: A review. Adv. Colloid Interface Sci..

[59] Matei A.-M., Caruntu C., Tampa M., Georgescu S.R., Matei C., Constantin M.M., Constantin T.V., Calina D., Ciubotaru D.A., Badarau I.A. (2021). Applications of Nanosized-Lipid-Based Drug Delivery Systems in Wound Care. Appl. Sci..

[60] Taheri M., Arabestani M.R., Kalhori F., Asl S.S., Asgari M., Hosseini S.M. (2024). Antibiotics-encapsulated nanoparticles as an antimicrobial agent in the treatment of wound infection. Front. Immunol..

[61] Hou X., Zaks T., Langer R., Dong Y. (2021). Lipid nanoparticles for mRNA delivery. Nat. Rev. Mater..

[62] Franco D., Calabrese G., Guglielmino S.P.P., Conoci S. (2022). Metal-Based Nanoparticles: Antibacterial Mechanisms and Biomedical Application. Microorganisms.

[63] Arana L., Gallego L., Alkorta I. (2021). Incorporation of Antibiotics into Solid Lipid Nanoparticles: A Promising Approach to Reduce Antibiotic Resistance Emergence. Nanomaterials.

[64] Thorn C.R., Thomas N., Boyd B.J., Prestidge C.A. (2021). Nano-fats for bugs: The benefits of lipid nanoparticles for antimicrobial therapy. Drug Deliv. Transl. Res..

[65] Thi T.T.H., Suys E.J.A., Lee J.S., Nguyen D.H., Park K.D., Truong N.P. (2021). Lipid-Based Nanoparticles in the Clinic and Clinical Trials: From Cancer Nanomedicine to COVID-19 Vaccines. Vaccines.

[66] Solidum J.G.N., Ceriales J.A., Ong E.P., Ornos E.D.B., Relador R.J.L., Quebral E.P.B., Lapeña J.F.F., Tantengco O.A.G., Lee K.Y. (2023). Nanomedicine and nanoparticle-based delivery systems in plastic and reconstructive surgery. Maxillofac. Plast. Reconstr. Surg..

[67] Thakur K., Sharma G., Singh B., Chhibber S., Katare O.P. (2019). Nano-engineered lipid-polymer hybrid nanoparticles of fusidic acid: An investigative study on dermatokinetics profile and MRSA-infected burn wound model. Drug Deliv. Transl. Res..

[68] Zhou Y., Cai C.-Y., Wang C., Hu G.-M., Li Y.-T., Han M.-J., Hu S., Cheng P. (2023). Ferric-loaded lipid nanoparticles inducing ferroptosis-like cell death for antibacterial wound healing. Drug Deliv..

[69] Ryan A., Patel P., Ratrey P., O’connor P.M., O’sullivan J., Ross R.P., Hill C., Hudson S.P. (2023). The development of a solid lipid nanoparticle (SLN)-based lacticin 3147 hydrogel for the treatment of wound infections. Drug Deliv. Transl. Res..

[70] Zielińska A., Carreiró F., Oliveira A.M., Neves A., Pires B., Venkatesh D.N., Durazzo A., Lucarini M., Eder P., Silva A.M. (2020). Polymeric Nanoparticles: Production, Characterization, Toxicology and Ecotoxicology. Molecules.

[71] Deng S., Gigliobianco M.R., Censi R., Di Martino P. (2020). Polymeric Nanocapsules as Nanotechnological Alternative for Drug Delivery System: Current Status, Challenges and Opportunities. Nanomaterials.

[72] Begines B., Ortiz T., Pérez-Aranda M., Martínez G., Merinero M., Argüelles-Arias F., Alcudia A. (2020). Polymeric Nanoparticles for Drug Delivery: Recent Developments and Future Prospects. Nanomaterials.

[73] Cano A., Ettcheto M., Espina M., López-Machado A., Cajal Y., Rabanal F., Sánchez-López E., Camins A., García M.L., Souto E.B. (2020). State-of-the-art polymeric nanoparticles as promising therapeutic tools against human bacterial infections. J. Nanobiotechnol..

[74] Kamaruzzaman N.F., Tan L.P., Hamdan R.H., Choong S.S., Wong W.K., Gibson A.J., Chivu A., Pina M.D.F. (2019). Antimicrobial Polymers: The Potential Replacement of Existing Antibiotics?. Int. J. Mol. Sci..

[75] Namiot E.D., Sokolov A.V., Chubarev V.N., Tarasov V.V., Schiöth H.B. (2023). Nanoparticles in Clinical Trials: Analysis of Clinical Trials, FDA Approvals and Use for COVID-19 Vaccines. Int. J. Mol. Sci..

[76] Hasan N., Cao J., Lee J., Hlaing S.P., Oshi M.A., Naeem M., Ki M.-H., Lee B.L., Jung Y., Yoo J.-W. (2019). Bacteria-Targeted Clindamycin Loaded Polymeric Nanoparticles: Effect of Surface Charge on Nanoparticle Adhesion to MRSA, Antibacterial Activity, and Wound Healing. Pharmaceutics.

[77] Matica M.A., Aachmann F.L., Tøndervik A., Sletta H., Ostafe V. (2019). Chitosan as a Wound Dressing Starting Material: Antimicrobial Properties and Mode of Action. Int. J. Mol. Sci..

[78] Alven S., Aderibigbe B.A. (2020). Chitosan and Cellulose-Based Hydrogels for Wound Management. Int. J. Mol. Sci..

[79] Delli Santi G., Borgognone A. (2019). The use of Epiprotect®, an advanced wound dressing, to heal paediatric patients with burns: A pilot study. Burn. Open.

[80] Naskar A., Kim K.-S. (2020). Recent Advances in Nanomaterial-Based Wound-Healing Therapeutics. Pharmaceutics.

[81] Anisha B., Biswas R., Chennazhi K., Jayakumar R. (2013). Chitosan–hyaluronic acid/nano silver composite sponges for drug resistant bacteria infected diabetic wounds. Int. J. Biol. Macromol..

[82] Wolfram J., Zhu M., Yang Y., Shen J., Gentile E., Paolino D., Fresta M., Nie G., Chen C., Shen H. (2015). Safety of Nanoparticles in Medicine. Curr. Drug Targets.

[83] Xuan L., Ju Z., Skonieczna M., Zhou P., Huang R. (2023). Nanoparticles-induced potential toxicity on human health: Applications, toxicity mechanisms, and evaluation models. Medcomm.

[84] Egbuna C., Parmar V.K., Jeevanandam J., Ezzat S.M., Patrick-Iwuanyanwu K.C., Adetunji C.O., Khan J., Onyeike E.N., Uche C.Z., Akram M. (2021). Toxicity of Nanoparticles in Biomedical Application: Nanotoxicology. J. Toxicol..

[85] Tian L., Lin B., Wu L., Li K., Liu H., Yan J., Liu X., Xi Z. (2015). Neurotoxicity induced by zinc oxide nanoparticles: Age-related differences and interaction. Sci. Rep..

[86] Awasthi S., Srivastava A., Kumar D., Pandey S.K., Mubarak N.M., Dehghani M.H., Ansari K. (2024). An insight into the toxicological impacts of carbon nanotubes (CNTs) on human health: A review. Environ. Adv..

[87] Møller P., Wils R.S., Di Ianni E., Gutierrez C.A.T., Roursgaard M., Jacobsen N.R. (2021). Genotoxicity of multi-walled carbon nanotube reference materials in mammalian cells and animals. Mutat. Res. Mol. Mech. Mutagen..

[88] Svadlakova T., Holmannova D., Kolackova M., Malkova A., Krejsek J., Fiala Z. (2022). Immunotoxicity of Carbon-Based Nanomaterials, Starring Phagocytes. Int. J. Mol. Sci..

[89] Sharma A., Madhunapantula S.V., Robertson G.P. (2012). Toxicological considerations when creating nanoparticle-based drugs and drug delivery systems. Expert Opin. Drug Metab. Toxicol..

